# Berkchaetoazaphilone B has antimicrobial activity and affects energy metabolism

**DOI:** 10.1038/s41598-021-98252-w

**Published:** 2021-09-21

**Authors:** Xudong Ouyang, Jelmer Hoeksma, Gisela van der Velden, Wouter A. G. Beenker, Maria H. van Triest, Boudewijn M. T. Burgering, Jeroen den Hertog

**Affiliations:** 1 Hubrecht Institute-KNAW and University Medical Center Utrecht, Utrecht, The Netherlands; 2 Institute Biology Leiden, Leiden University, Leiden, The Netherlands; 3 Oncode Institute and Molecular Cancer Research, Center Molecular Medicine, University Medical Center Utrecht, Utrecht, The Netherlands

**Keywords:** Fungi, Antibiotics, Natural products

## Abstract

Antimicrobial resistance has become one of the major threats to human health. Therefore, there is a strong need for novel antimicrobials with new mechanisms of action. The kingdom of fungi is an excellent source of antimicrobials for this purpose because it encompasses countless fungal species that harbor unusual metabolic pathways. Previously, we have established a library of secondary metabolites from 10,207 strains of fungi. Here, we screened for antimicrobial activity of the library against seven pathogenic bacterial strains and investigated the identity of the active compounds using ethyl acetate extraction, activity-directed purification using HPLC fractionation and chemical analyses. We initially found 280 antimicrobial strains and subsequently identified 17 structurally distinct compounds from 26 strains upon further analysis. All but one of these compounds, berkchaetoazaphilone B (BAB), were known to have antimicrobial activity. Here, we studied the antimicrobial properties of BAB, and found that BAB affected energy metabolism in both prokaryotic and eukaryotic cells. We conclude that fungi are a rich source of chemically diverse secondary metabolites with antimicrobial activity.

## Introduction

In the pre-antibiotic era, the average life expectancy was around 47 years, partly because of the high mortality due to infectious diseases^1^. This situation was changed by the discovery of penicillin in 1928, which innovated the course of medicine and was marked as the beginning of the antibiotic era^2^. However, an issue arose in connection to the use of antibiotics, antimicrobial resistance^3–5^. Although many classes of antimicrobials have been discovered, resistance to these antimicrobials developed shortly after their use in hospitals^6^. Bacterial infection has become a serious threat to humans again because of this emerging resistance, especially when the so-called “superbugs”, which are pathogens that are resistant to multiple antibiotics, appear in hospitals^7^. The treatment of infections is becoming complex, resulting in rising costs and higher patient mortality. In Europe, around 25,000 deaths are associated with antimicrobial resistance annually, costing 1.5 billion euros each year^8,9^. Thus, discovering novel antimicrobials with a new mechanism of action (MoA) is an endless task to fight newly emerging resistance.

The kingdom of fungi provides a wealth of antimicrobial agents^10^. There are countless fungal species that harbor unusual metabolic pathways^11^. The chemical products of these pathways are termed secondary metabolites, which are not required for life and growth of fungi per se. Secondary metabolites are often secreted bioactive compounds with low-molecular-weight that are produced at specific stages of morphological differentiation to develop interactions with other organisms or the environment^12^. Over millions of years, fungi have evolved in part by production of metabolites to perform important functions, to survive in harsh environments, to fight off invaders or to alter fungal development^13–16^. Thus, the functionally distinct fungal secondary metabolites have formed a diverse pool of biologically active natural compounds, including ones that are harmful (e.g. toxins) or beneficial (e.g. antimicrobials) to human beings^14^, which is promising for drug discovery.

The systematic study of fungal secondary metabolites started in 1922 when Harold Raistrick identified over 200 metabolites^17^. Unfortunately, it did not attract much attention from the public. This situation changed in 1928 with the discovery of penicillin. Since then, studies into fungal secondary metabolites have been conducted, and thousands of metabolites with antimicrobial, antifungal or antitumor activities have been discovered^11^. Compared to the number of existing fungal species^18^, this was only the tip of the iceberg. Due to the recent development of genome sequencing and bioinformatic analysis, genome mining has become a popular technique to screen for biosynthetic gene clusters of fungal secondary metabolites^19^. However, genome data are not always available for the less studied species, which have a high chance to produce metabolites that have not been described before. The traditional cultivation-based method facilitates analysis of these species.

Previously, a library of fungal secondary metabolites from 10,207 strains was established in our lab, and zebrafish (*Danio rerio*) embryos were used as read-out to screen for novel bioactive compounds that induced developmental defects^20^. Over 30 compounds including many relatively unexplored bioactive compounds were successfully identified from that screen. Here, we applied this library to screen for novel antimicrobial compounds. To this end, we screened antimicrobial activity against seven pathogenic bacterial strains. Next, we purified the active compounds using ethyl acetate extraction and HPLC fractionation and identified them using chemical analysis. The identified metabolites consist of both known antimicrobial compounds as well as relatively unexplored compounds. One of these compounds was berkchaetoazaphilone B (BAB), an anti-cancer compound found in 2015^21^, which was identified to contain antimicrobial activity in this screen.

## Materials and methods

### Strains, reagents and cultures

Pathogenic bacteria were obtained from University Medical Center Utrecht and are listed in Table S1. *Bacillus subtilis* 168 was used to test antimicrobial activity in this study^22^. Fungal strains were obtained from Westerdijk Fungal Biodiversity Institute and were inoculated for 7 days on Malt Extract Agar (MEA) plates. For liquid cultures, two cubes of agar with surface area of approximately 0.25 cm^2^ from each fungal species were cut and transferred into a 100 mL bottle containing 50 mL culturing medium (3.5% Czapek Dox Broth (CDB) + 0.5% yeast extract (YE)). Cultures were incubated at 25 °C for 7–14 days (depending on their growth) and were then filtered using 0.22 µm Millipore filters. For plate cultures, two cubes of agar from each fungal species were cut and transferred onto new specific agar plates and incubated at 25 °C for 7–14 days, depending on their growth. Commercial antimicrobials and resazurin were purchased from Sigma Aldrich. FM4-64 and DiSC_3_(5) were purchased from Thermo Fisher Scientific.

### Antimicrobial activity screening

Bacterial cultures of different strains were grown to early exponential-phase in Mueller Hinton Broth (MHB) from their overnight cultures. Then they were diluted 1:100 into MHB, distributed into 96-well plates and tested with 1:1 ratio of fungal supernatants. Inhibition of bacterial growth was checked based on visual inspection after an overnight incubation at 37 °C. Antimicrobial activities and maximum inhibitory dilution (MID) were determined by testing of a range of fungal supernatant dilutions in a broth microdilution assay^23^. MID was defined as the highest dilution at which bacteria did not grow, based on visual inspection after an overnight incubation at 37 °C.

### Purification and identification of biologically active compounds

Liquid cultures or agar plates were extracted with ethyl acetate. The solvent was evaporated and the residue was dissolved in DMSO. Extracts were fractionated using a modular preparative high-performance liquid chromatography (HPLC) system (Shimadzu) using a C18 reversed phase Reprosil column (10 μm, 120 Å, 250 × 22 mm). The mobile phase was 0.1% trifluoroacetic acid in water (buffer A) and 0.1% trifluoroacetic acid in acetonitrile (buffer B) in a linear gradient. Fractions were collected, dried in an Eppendorf speedvac concentrator, dissolved in DMSO and tested for antimicrobial activities.

The identification procedure was performed as previously described^20^ using preparative HPLC (Shimadzu) using a Shimadzu Shim-pack GISTC18-HP reversed phase column (3 μm, 4.6 × 100 mm) coupled to a LCMS-2020 mass spectrometer (Shimadzu). High resolution mass spectrometry (HRMS) was measured on either a µQTOF instrument (Micromass Ltd) or an LCT instrument (Micromass Ltd). Samples were dissolved in DMSO-d_6_ or CDCl_3_ for NMR spectroscopy. ^1^H-NMR, HSQC, HMBC and COSY spectra were measured at either 300 MHz, 400 MHz, 500 MHz or 600 MHz using either a Mercury-300, an Agilent-400, an INOVA-500 or a Bruker-600 spectometer. ^13^C-NMR was measured using the same instruments at either 100 MHz or in case the Bruker-600 instrument was used at 150 MHz.

### Growth curves

The overnight cultures of *B. subtilis* were diluted 1:50 into fresh LB medium and incubated at 37 °C with shaking. OD_600_ of cultures were measured by a FLUOstar microplate reader (BMG Labtech) every 30 min for 24 h. At an OD_600_ of 0.3, different concentrations of BAB were added.

### Confocal microscopy

Microscopy was performed using a Perkin Elmer UltraView VoX spinning disk microscope essentially as described^24^ and analyzed using Volocity v6.3 software. Z-stack images were collected over a length of 3 µm with 0.2 µm intervals and analyzed using Fiji^25^.

### Sporulation inhibition assay

Sporulation was assayed as previously described with modifications^26^. Antimicrobials (5 × minimal inhibitory concentration, MIC) or DMSO (control) were added and incubated with rolling at 37 °C for 5 h. Cells were then stained with FM 4–64, immobilized and imaged.

### Resazurin assay

This resazurin oxidation–reduction indicator^27^ assay was done as previously described^24^. Freshly prepared early exponential-phase cell cultures with an OD_600_ of 0.3 in LB medium were treated with antimicrobials (5 × MIC) or DMSO (control) for 5, 20 or 60 min. Cells were then incubated with 30 µg/mL resazurin for 45 min at 37 °C. Cells without any agent and boiled cells (95 °C for 10 min) were used to calculate the standard respiration and no respiration, respectively. Absorbance of different samples was measured using a 540 nm/590 nm filter.

### Screen for hypersensitive *S. aureus* transposon mutants to BAB with Nebraska Transposon Mutant Library (NTML)

The NTML consists of 1920 *S. aureus* transposon mutants. The strains were inoculated with 100 µL MHB containing 5 µg/mL erythromycin in sterile round-bottom 96-wells plates at 37 °C with shaking overnight. On the second day, 5 µL overnight culture was transferred into 95 µL MHB containing 5 µg/mL erythromycin for 2 h to make the start culture. Next, 5 µL start culture was added into 95 µL fresh MHB containing an antimicrobial of interest. After incubation overnight at 37 °C with shaking, bacterial growth was inspected visually.

### Cytotoxicity assay

HepG2 cells were seeded in 96-well plates and grown in DMEM low glucose medium (ThermoFisher) supplemented with 10% FBS. Test compounds were added in different concentrations, with a final concentration of 1% DMSO and cells were incubated for 20 h at 37 °C with 5% CO_2_. Next, resazurin (Sigma-Aldrich) was added to reach a final concentration of 0.1 mM. After 3 h incubation, the fluorescence was measured on a PHERAstar microplate reader (BMG Labtech). Experiments were conducted in biological triplicates. IC_50_ was calculated using nonlinear regression in GraphPad Prism.

### Measuring bioenergetics using Seahorse

Seahorse Bioscience XFe24 Analyzer was used to measure extracellular acidification rates (ECAR) in mpH/minute and oxygen consumption rates (OCR) in pmol O_2_/minute essentially as described before^28^. Cells were seeded in XF24 polystyrene cell culture microplates (Seahorse Bioscience) at a density of 20,000 cells per well. One hour before the measurements, culture medium was replaced either with or without BAB and the plate was incubated for 60 min at 37 °C. For the mitochondrial stress test, culture medium was replaced by Seahorse XF Base medium (Seahorse Bioscience), supplemented with 20 mM glucose (Sigma-Aldrich), 2 mM L-glutamine (Sigma-Aldrich), 5 mM pyruvate (Sigma-Aldrich) and 0.56 μL NaOH (1 M). During the test 5 μM oligomycin, 2 μM FCCP and 1 μM of Rotenone and Antimycin A (all Sigma-Aldrich) were injected into each well after 18, 45 and 63 min respectively. For the glycolysis stress test, culture medium was replaced by Seahorse XF Base medium, supplemented with 2 mM L-glutamine and 0.52 μL/mL NaOH (1 M). Sensor cartridges (pre‐hydrated in XF calibrant solution overnight in a CO_2_‐free incubator) were loaded with glucose (Port A), oligomycin (Port B), and 2‐deoxyglucose (2-DG, Port C) to achieve concentrations of 1 mM, 2 μM, and 50 mM, respectively, after injection. During the test 10 mM glucose, 5 μM oligomycin and 100 mM 2-deoxyglucose (2-DG) (Sigma-Aldrich) were injected into each well after 18, 36 and 65 min respectively. After injections, measurements of 2 min were performed in triplicate, preceded by 4 min of mixture time. The first measurements after oligomycin injections were preceded by 5 min mixture time, followed by 8 min waiting time for the mitochondrial stress test and 5 min mixture time followed by 10 min waiting time for the glycolysis stress test. Both ECAR and OCR were normalized to individual protein amount, and data were analysed using the XF Mito Stress Test Report Generator.

## Results

### In search of antimicrobials from a fungal metabolites library

**Figure 1:**
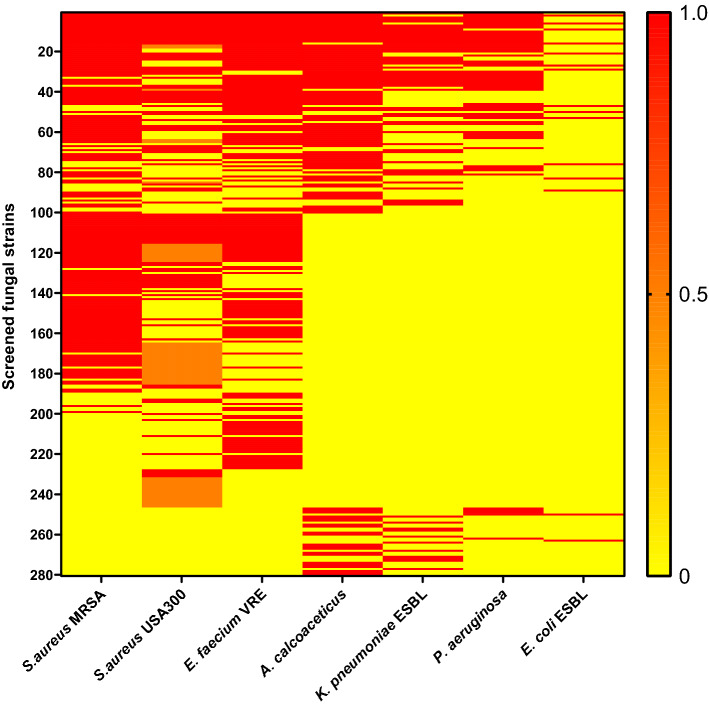
Initial screen for antimicrobial activity from fungal secondary metabolites library. Each row of the map shows the activity of a single fungus against seven different bacteria. Active, half-active and inactive are indicated as “1” (red), “0.5” (orange) and “0” (yellow), respectively.

To assess the antimicrobial activity of the fungal metabolite library, an initial screen was done by testing all the 10,207 fungal supernatants on seven pathogenic bacteria, including three Gram-positive strains and four Gram-negative strains. To determine their activity, log-phase bacteria were grown overnight in the presence of fungal supernatant. Subsequently, the inhibitory effect was scored by visual inspection. A total inhibition was recorded as “1” and no inhibition as “0”. For some of the hits against *Staphylococcus aureus* USA300, bacterial growth was affected but not 100% inhibited. For these, a number of “0.5” was recorded. In the end, 280 fungi (2.7%) were defined as antimicrobial strains and listed in Fig. 1 as they showed antimicrobial activity against at least one of the bacterial strains. Among all tested pathogenic bacteria, the greatest number of hits was found to inhibit the growth of *S. aureus*. There were more than 140 hits against each of the two pathogenic *S. aureus* strains we tested. In contrast, the extended spectrum beta-lactamase (ESBL) producing *Escherichia coli* was the most difficult pathogen to target in our assays, as we found only 15 hits.

Of the 280 fungi with potential antimicrobial activity, 36% (100 strains) showed activity against both Gram-positive and Gram-negative bacteria. No fungus inhibited all seven pathogens but 39 fungi were able to affect the growth of five or six bacterial strains, including 28 fungi from the genera of *Aspergillus* and *Penicillium*. The species from these two genera are well studied producers of antimicrobial compounds, which provided proof-of-principle for the method we applied to uncover antimicrobial producing fungi. The other 180 strains showed activity only against Gram-positive or Gram-negative bacteria. In line with the notion that Gram-positive bacteria are more sensitive to antimicrobials than Gram-negative bacteria^29^, 146 strains (52% of the total hits) only affected the growth of Gram-positive bacteria and 34 strains (12% of the total) only inhibited growth of Gram-negative bacteria.

As expected, there were multiple well-known producers of antimicrobial agents among the potential hits. To increase the chances to identify compounds that had not been described before, we focused on poorly studied fungi of which no information was available about secondary metabolites. We selected 56 fungal strains in this category and investigated their antimicrobial activities in detail.

### Identification strategy of antimicrobial compounds

**Figure 2:**
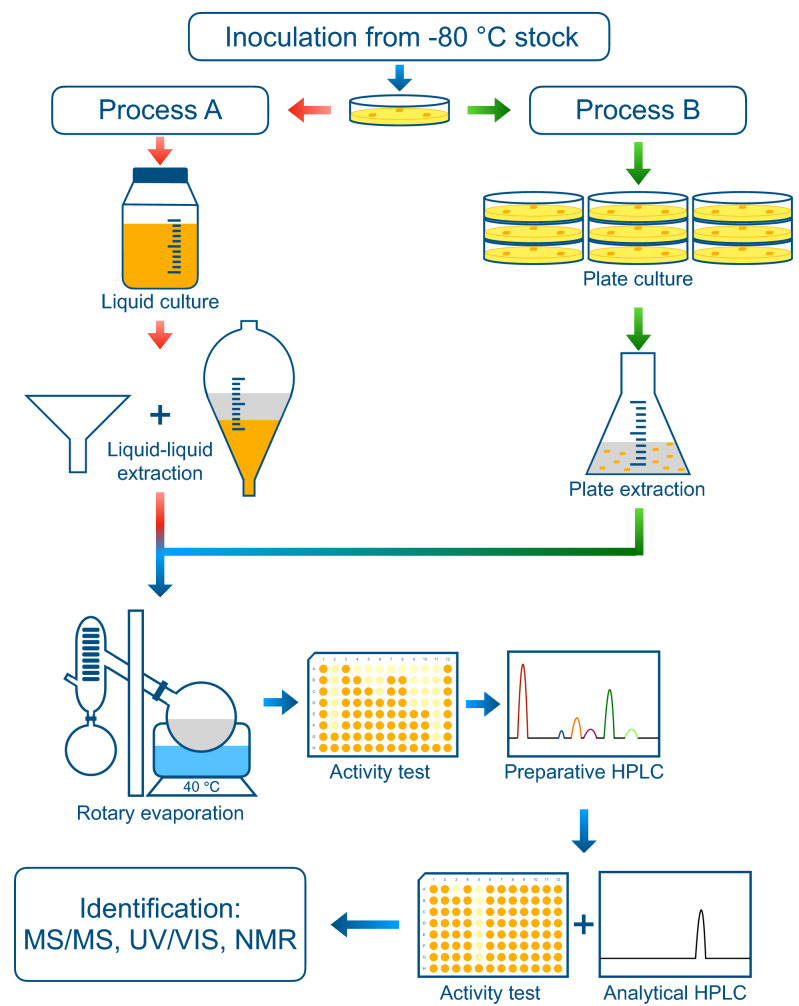
Identification strategy of antimicrobial compounds from fungi. Fungi were inoculated on agar plates and subsequently cultured in liquid media, filtrated by 0.22 µm filter, and extracted with ethyl acetate as shown in Process A (red arrows). Next, samples were concentrated using a rotary evaporator and tested for their antimicrobial activity in 96-well plates. Active samples were fractionated by preparative-HPLC, followed by activity (96-well plates) and purity (analytical HPLC) check. Pure active fractions were then identified by a combination of several chemical analyses. If the yield of active compounds was not sufficient by culturing in liquid medium, plate extraction (Process B, green arrows) was applied by culturing fungi on agar plates and extracting compounds with ethyl acetate directly from cultures on agar.

The secondary metabolites responsible for the observed antimicrobial activities were isolated and identified, using an activity-guided purification and identification procedure as outlined in Fig. 2 (Process A). First, to increase the yield of fungal compounds, we cultured 1 L of each fungus in liquid medium and extracted metabolites from the liquid culture with ethyl acetate. The extracts were dried using a rotary evaporator and dissolved in DMSO.

**Figure 3:**
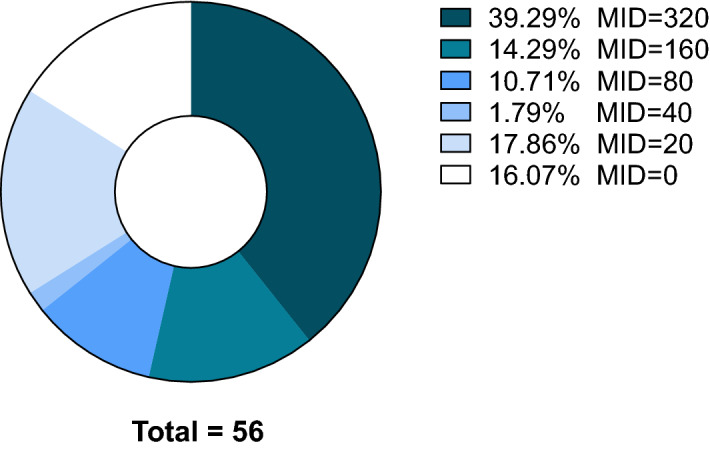
Re-screen of antimicrobial activity from 56 hits. The extracts from 56 fungi were tested for their maximum inhibitory dilutions (MIDs) against *B. subtilis*. The MID of each fungus was plotted in a pie chart. Highest active dilution was 320 × diluted.

The widely used Gram-positive bacteria model, *B. subtilis* strain 168, was used as a read-out for further analysis of the antimicrobial activities and for analysis of the MoA of selected antimicrobial agents. The ethyl acetate extracts were tested on *B. subtilis* using microdilution assay until 320 × diluted and the maximum inhibitory dilution (MID) was recorded as the criterion of the activity. In the end, 47 extracts (around 84% of the strains, Fig. 3) showed antimicrobial activity. Of these, 22 strains showed MIDs greater or equal to 320, 8 showed MIDs equal to 160, and 17 showed MIDs less than 100. These 47 extracts were fractionated through preparative HPLC to obtain pure compounds. The resulting fractions, each containing a single HPLC peak were then tested on *B. subtilis*. For the chemical analysis, we first determined the purity, UV/VIS spectra and nominal mass of the active fractions using analytical HPLC and LC–MS. Next, we compared the resulting data with available literature and databases^30,31^, through which we were able to identify several compounds from active fractions as previously identified compounds. For the remaining unsolved fractions, accurate mass was determined through high resolution mass spectrometry (HRMS) and structural information was obtained using ^1^H-NMR, ^13^C-NMR and 2D-NMR.

Through activity-directed purification as described above, the antimicrobial agents from 13 fungi were successfully identified (A1 in Table 1). For the other strains, the amounts of active compound(s) were not sufficient for chemical analysis. Therefore, we sought to increase the yield of secondary metabolites through optimization.

**Table 1:** Fungal stains with identified metabolites.

CBS number	Species	MID	Process A or B	Metabolites
CBS 111.69	*Corynascus sepedonium*	320	A1	UCS-1025A
CBS 180.74	*Sarocladium oryzae*	320	A1	Helvolic acid
CBS 194.67	*Ulocladium atrum*	160	A2	Dehydrocurvularin
CBS 245.59	*Fusarium sacchari*	320	A1	Fusaric acid
CBS 279.58	*Paecilomyces lilacinus*	80	A2	Leucinostatins
CBS 305.72	*Trichophaea abundans*	80	A2	Anthracobic acid A
CBS 316.67	*Eupenicillium senticosum*	320	A1	Dehydrocurvularin
CBS 348.82	*Dactylaria lanosa*	80	A2	Norlichexanthone
CBS 349.73	*Monochaetia lutea*	320	B	Rugulosin A
CBS 366.71	*Oidiodendron flavum*	40	B	Harzianic acid
CBS 399.73	*Sarocladium attenuatum*	320	A1	Helvolic acid
CBS 417.64	*Mortierella globulifera*	320	A1	Leucinostatins
CBS 438.86	*Arthroderma tuberculatum*	320	A1	Fusidic acid
CBS 511.67	*Stilbella fimetaria*	320	A1	Helvolic acid
CBS 573.67	*Pleurostomophora richardsiae*	320	B	Berkchaetoazaphilone B
CBS 668.70	*Clonostachys compactiuscula*	160	B	TMC-154
CBS 114,383	*Corynascus sepedonium*	320	A1	UCS-1025A
CBS 124,389	*Cristaspora arxii*	160	B	Gliotoxin
/	Unknown species 2121	320	B	Asterric acid, 4'-chloroasterric acid, geodin hydrate
/	Unknown species 2172	320	A1	Citrinin
/	Unknown species 2212	320	A1	Citrinin
/	Unknown species 2239	80	A2	Citrinin
/	Unknown species 2287	80	A2	Citrinin
/	Unknown species 2288	320	A1	Leucinostatins
/	Unknown species 2998	160	B	Rugulosin A
/	Unknown species 9806	320	A1	Helvolic acid

The 26 selected fungal strains with identified activities are listed. The initial activity of their fungal liquid–liquid extracts (in maximum inhibitory dilution, MID) and the identification process that was used (A1 means from Process A without culturing optimization; A2 means Process A with culturing optimization; and B means from Process B) were also present. The strains without CBS numbers were a gift from the Westerdijk fungal biodiversity institute.

### Optimization for yield of fungal secondary metabolites

Environmental factors such as temperature, oxygen, humidity and nutrients are important for fungal metabolic pathways^32–34^. Here, we optimized four aspects of the culturing conditions: temperature, oxygen content, liquid growth medium and inoculating agar plates. Each of these aspects influenced the production of antimicrobial activity to a different extent in different fungi (examples in Table S2). In the end, the antimicrobial compounds from six more fungi were identified (A2 in Table 1). Surprisingly, some fungal species, like the case of *Monochaetia lutea*, showed remarkable morphological differences on different inoculation plates (Fig. S1), but the activity of the respective liquid cultures did not differ much (Table S2). To study the differences further, we set up an alternative strategy to obtain secondary metabolites, which was to extract compounds directly from agar plates, as outlined in Fig. 2 (Process B). In practice, we compared agar-extracts from fungi grown on different plates using analytical HPLC and microdilution assay, to select the best conditions for large-scale cultures. Using the plate extraction strategy, the active compounds from seven fungi were successfully identified (Table 1).

### Identification of BAB from *Pleurostomophora richardsiae*

**Figure 4:**
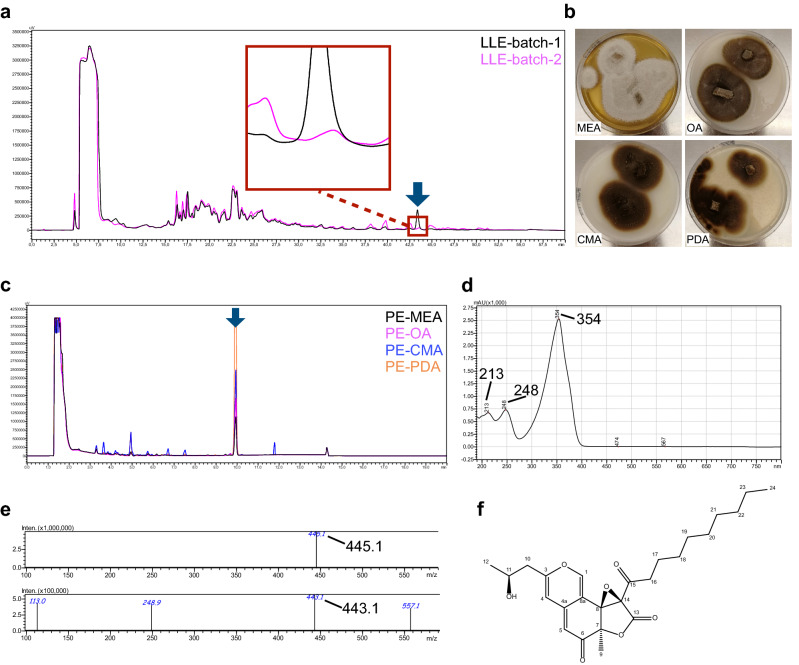
Identification of the antimicrobial activity from fungus *Pleurostomophora richardsiae*. (**a**) Preparative HPLC profiles of extracts from two batches of liquid culture using liquid–liquid extraction (LLE). (**b**) *P. richardsiae* cultured on different kinds of agar. (**c**) Comparison of different plate extractions (PE) on analytical HPLC. (**d**) UV spectrum of the active compound from this fungus. (**e**) Mass spectrum of the active compound. (**f**) Chemical structure of BAB, the antimicrobial activity from *P. richardsiae*.

The supernatant of *P. richardsiae* was shown to have antimicrobial activity in the initial screen and the active fraction was determined in the re-screen. However, the activity appeared to vary across different batches of liquid cultures (Fig. 4a). To optimize the production of the active fraction, we extracted the secondary metabolites from plate cultures (Fig. 4b). Analytical HPLC analysis of plate extracts from different agars showed that potato dextrose agar (PDA) plates induced the highest yield of the active compound (Fig. 4c). Subsequently, we cultured this fungus on 20 PDA plates and obtained sufficient amounts of the active fraction for its identification using chemical analyses.

First, the active fraction was measured on an LC–MS with diode array detection to assess the purity and meanwhile obtain a UV–Vis spectrum. The UV–Vis spectrum showed a distinct pattern with maximum absorption at 354 nm and lower peaks at 248 nm and 213 nm (Fig. 4d). The mass spectra revealed a M + H ion signal of 445.1 in the positive spectrum and a M-H ion signal of 443.1 in the negative spectrum (Fig. 4e), indicating a nominal mass of 444 for the main compound of the fraction. Next, HRMS was performed on the active compound and a mass of 467.2060 was found for the M + Na ion. Finally, nuclear magnetic resonance (NMR) spectra were collected using ^1^H, ^13^C, Heteronuclear Single-Quantum Correlation Spectroscopy (HSQC), Heteronuclear Multiple-Bond Correlation spectroscopy (HMBC) and homonuclear correlation (COSY) (Table S3, Fig. S2). All data combined indicated that this compound was BAB, previously published by Stierle et al^21^.

*Berkchaetoazaphilone B* (Fig. 4f): C_25_H_32_O_7_. HRMS: found 467.2060 (M + Na), calculated 467.2046 for C_25_H_32_O_7_Na. NMR (400 MHz, DMSO-d_6_): see Table S3. UV–Vis λ_max_: 213 nm, 248 nm, 354 nm.

### Fungi produced a variety of structurally different compounds with antimicrobial activity

Using our antimicrobial identification strategy, 17 antimicrobial agents were successfully identified from 26 fungi (Table 1, Fig. 4f, Fig. S3) and the chemical data that were used to identify compounds are listed in the Supplementary Material. Previously, we found that two of these compounds, fusaric acid and anthracobic acid also induced developmental defects in a zebrafish screen and we have described these compounds before^20^. Some of the antimicrobial compounds that we identified were reported to have other kinds of activities, including immunosuppressive activity of gliotoxin^35^, anti-osteoporosis activity of norlichexanthone^36^ and plant promoting activity of harzianic acid^37^. Structurally, these fungal secondary metabolites belong to a variety of chemical groups including polyketides (e.g. TMC-154, citrinin and BAB), lactones (e.g. dehydrocurvularin), lipopeptides (e.g. leucinostatins), terpenoids (e.g. helvolic acid) and piperazines (e.g. gliotoxin), suggesting our strategy was effective for purification and identification of diverse chemical groups.

A noteworthy compound we found in our screen is BAB, which was initially identified and described as a cytotoxin towards human cancer cells in 2015^21^. No data were available about its antimicrobial activity. Therefore, we investigated its antimicrobial properties.

### BAB has antimicrobial activity against gram-positive bacteria

**Figure 5:**
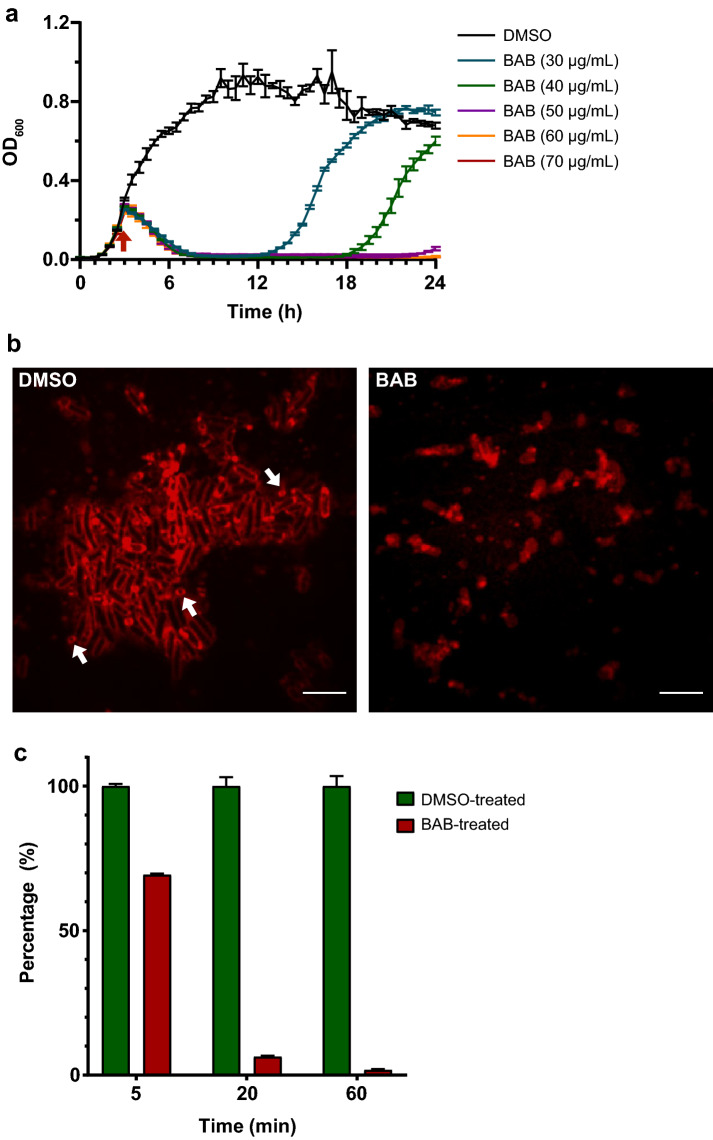
Antimicrobial properties of BAB. (**a**) Growth curves of *B. subtilis* in the presence of a range of BAB concentrations. OD_600_ was measured every 30 min. BAB was added at 2 h 45 min (arrow indicated). The graph depicts the average and the SEM of biological triplicates. (**b**) No sporulation in response to BAB. *B. subtilis* cells from high intensity overnight culture were treated with DMSO (control) or BAB (250 μg/mL, 5 × MIC), stained with FM4-64 and imaged by confocal fluorescence microscopy. Representative images are shown. Example spores in the DMSO control are indicated with arrows. Scale bar is 5 µm. (**c**) Effect on respiratory chain activity measured by the reduction from blue resazurin to red resorufin at 540 nm. The average intensity of DMSO control in each group was set as 100% intensity and the percentage of each treated sample was calculated. The mean from biological triplicates was plotted with error bars representing the SEM.

First, to determine the antimicrobial spectrum of BAB, we tested its minimum inhibitory concentration (MIC) on 13 bacterial strains (Table S4). Most Gram-positive bacteria were inhibited at MICs between 50 and 200 μg/mL, including a vancomycin-resistant *Enterococcus faecium* (VRE) strain. However, BAB had no effect on any of the Gram-negative bacteria we tested. This suggested that BAB is a selective antimicrobial against Gram-positive bacteria. Therefore, we chose a model organism of Gram-positive bacteria, *B. subtilis* strain 168, to further describe its antimicrobial property. The growth curves of *B. subtilis* in response to a concentration range of BAB showed that bacterial growth was affected from 30 μg/mL onwards, and that growth was completely arrested from 50 μg/mL onwards (Fig. 5a). The OD_600_ decreased at 30 μg/mL and higher, suggesting that BAB might induce cell lysis.

Some microorganisms, including *B. subtilis*, respond to harsh environments by entering a robust resting state, the endospore, which is self-protective. During that process, cells may also lyse^38^. To determine whether BAB destroyed the cells and/or induced the cells to form endospores, a sporulation assay was performed (Fig. 5b). In the control-treated samples, spores were clearly observed in the overnight culture with high cell density. Following BAB treatment, only cell debris was evident in the micrographs. This suggests that BAB destroyed the cells without inducing spore formation.

To determine if the cell lysis was due to a direct target of BAB in the cell membrane, a cell depolarization assay was performed using DiSC_3_(5). Treatment with carbonyl cyanide m-chlorophenyl hydrazone (CCCP), a control compound that induces cell depolarization, led to an increase in fluorescence. Rifampin and DMSO treated samples were used as negative controls. Surprisingly, BAB treatment led to a sudden decrease (to an average of 33% of control) in fluorescence (Fig. S4a). However, the decrease in signal was due to a direct effect of BAB on DiSC_3_(5), because BAB also caused a decrease (to 33% of control) in fluorescence in cell-free medium (Fig. S4b). Therefore, interpretation of these data is hampered and we cannot conclude whether or not BAB affected cell depolarization.

Colorimetric analysis of respiratory chain activity using resazurin indicated that more than 90% of the cells had lost their viability at 20 min treatment (Fig. 5c). Taken together, BAB treatment inhibited bacterial growth in a dose-dependent manner and induced lysis of bacteria, but not sporulation. BAB treatment led to rapid respiratory chain activity arrest.

### BAB affects energy metabolism

To further determine the targets of BAB, we applied a sensitivity screen on Nebraska Transposon Mutant Library (NTML). This library contains approximately 2,000 *S. aureus* transposon mutants, each with a distinct disruption of a non-essential gene in the genome^39^. We screened for mutants that were more sensitive to BAB, because the corresponding genes might be essential to compensate for the defects caused by BAB. First, five random strains were selected to determine the MIC. Next, 0.5 × MIC was applied to each of the 1,920 strains and affected strains were selected. As listed in Table 2, the growth of 23 strains was inhibited in the first run. A MIC assay was performed to verify these hits, confirming that 16 strains indeed were hypersensitive to BAB and thus were regarded as potential hits.

**Table 2:** NTML screening suggests energy metabolism as target of BAB.

Number in NTML	Verification	Gene	Gene function	Pathway
NE91	Yes	kdpA	K + -transporting ATPase, A subunit	ATPase
NE353	Yes	/	Bifunctional purine biosynthesis protein	Purine biosynthesis
NE427	Yes	fumC	Fumarate hydratase, class II	Citric acid cycle
NE522	Yes	purB	Adenylosuccinate lyase	Purine biosynthesis
NE592	Yes	atpA	ATP synthase F1, alpha subunit	ATP synthesis
NE635	Yes	ribE	Riboflavin synthase, alpha subunit	Riboflavin synthesis
NE716	Yes	/	Putative membrane protein	/
NE744	/	/	/	/
NE950	/	/	/	/
NE1004	/	/	/	/
NE1016	Yes	/	Acetyltransferase	Citric acid cycle
NE1040	Yes	mutY	A/G-specific adenine glycosylase	DNA repair
NE1260	Yes	pckA	Phosphoenolpyruvate carboxykinase	Glycolysis
NE1263	Yes	mtlD	Mannitol-1-phosphate 5-dehydrogenase	Glycolysis
NE1318	Yes	ribH	6,7-dimethyl-8-ribityllumazine synthase	Riboflavin synthesis
NE1343	/	/	/	/
NE1345	/	/	/	/
NE1381	/	/	/	/
NE1494	Yes	rnc	Ribonuclease III	RNA synthesis
NE1569	Yes	/	Superantigen-like protein	Immune system
NE1757	Yes	lspA	Lipoprotein signal peptidase	/
NE1794	Yes	/	Holliday junction resolvase-like protein	DNA segregation
NE1833	/	/	/	/

0.5 × MIC of BAB was tested on each of the 1,920 strains and 23 inhibited strains were selected. These hits were verified with MIC assay and 16 strains indeed were hypersensitive to BAB. The mutated gene with its gene function and involved pathway for each of the 16 hits is presented.

Notably among these hits, half were involved in energy metabolism (Table 2, Fig. S5), including two hits in glycolysis, two in the citric acid cycle, one in purine biosynthesis, one in purine nucleotide cycle, one in ATP synthesis and one in the group of ATPases. The two riboflavin synthesis hits are also involved in energy metabolism. The remaining hits appeared not to have correlations with any of the other hits, or with each other. Based on these data, we hypothesized that BAB might affect energy metabolism.

**Figure 6:**
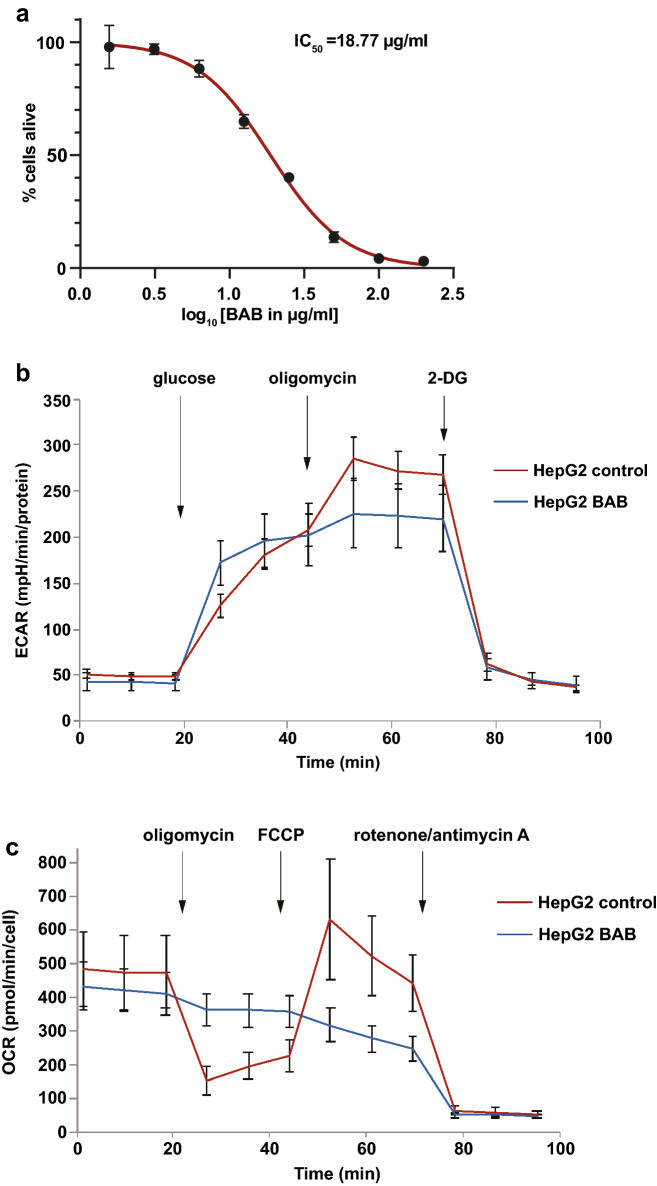
Cytotoxicity of BAB on HepG2 cells may be caused by effects on energy metabolism. (**a**) HepG2 cells with BAB in different concentrations were incubated for 20 h and afterwards resazurin was added. The ability to reduce blue resazurin to red resorufin was measured at 540 nm. The average intensity of DMSO control was set as 100% alive and the percentage of intensity from each treated sample was calculated. The mean from biological triplicates was plotted with error bars representing the SEM in black. Nonlinear regression was analyzed and plotted in red, on which IC_50_ was based. (**b**,**c**) HepG2 cells either untreated or pre-treated with BAB were compared in a glycolysis and mitochondrial test using Seahorse technology to measure extracellular acidification rates (ECAR) in mpH/minute and oxygen consumption rates (OCR) in pmol O_2_/minute. (**b**) For the glycolysis stress test, 10 mM glucose, 5 μM oligomycin and 100 mM 2-deoxyglucose (2-DG) were injected into each well after 18, 36 and 65 min respectively. (**c**) For the mitochondrial stress test, 5 μM oligomycin, 2 μM FCCP and 1 μM of Rotenone and Antimycin A were injected to each well after 18, 45 and 63 min respectively. Both ECAR and OCR were normalized to individual protein amount, and data from biological triplicates were presented by mean with error bars representing the SEM.

From our experiments, it is evident that BAB has bactericidal activity on Gram-positive bacteria. We assessed cytotoxicity of BAB on eukaryotic cells as well and found that BAB is cytotoxic for HepG2 cells with an IC50 of 18.77 μg/mL (Fig. 6a). Given the apparent involvement of BAB in energy metabolism of bacteria, we wondered whether BAB also targets energy metabolism in eukaryotic cells. To investigate this, we assayed the effect of BAB on bioenergetics of eukaryotic cells. We compared HepG2 cells either untreated or pre-treated with BAB in a glycolysis and mitochondrial test using Seahorse technology. BAB pre-treatment consistently resulted in a more rapid increase in glycolysis, yet reduced glycolytic capacity (Fig. 6b). This is likely linked to the clear impairment of mitochondrial function due to BAB pre-treatment. Pre-treatment with BAB resulted in a strong reduction in ATP forming capacity by mitochondria and an almost complete block of the carbonyl cyanide 4-(trifluoromethoxy)phenylhydrazone (FCCP)-induced increase in mitochondrial respiration, suggesting that BAB blocks either complex I or II or both (Fig. 6c). This inhibition of mitochondrial respiration and ATP formation likely explains increased glycolysis to uphold sufficient cellular ATP in BAB treated cells. Irrespective, these data clearly show that BAB has an effect on cellular bioenergetics, most profoundly on mitochondrial respiration, and this may underlie the mechanism of BAB.

## Discussion

Here, we screened for antimicrobial compounds from a fungal secondary metabolite library. In the end, the antimicrobial agents from almost half of the selected fungi were successfully identified. This suggests that our approach using ethyl acetate extraction (liquid–liquid extraction or plate extraction), reversed phase preparative-HPLC, analytical-HPLC, MS/MS and NMR can reliably be used for identification of a wide range of structurally different antimicrobial compounds. Ethyl acetate facilitates extraction of both polar and non-polar biological compounds^40^. However, in practice, highly polar compounds were not extracted efficiently, indicating that other approaches might be needed to extract these compounds.

All but one of the antimicrobial agents that we identified (Table 1) have been described before. Rediscovery of compounds is a widely acknowledged problem with activity-guided discovery approaches. BAB had not been reported to have antimicrobial activity. Previously, this compound was reported to have anti-cancer properties^21^. Our study showed that it had strong anti-bacterial activity against Gram-positive bacteria. By a series of bioactivity assays, we found that BAB rapidly blocked oxidation–reduction in bacteria, concomitant with cell lysis. Whether the cell envelope was targeted directly could not be concluded from the data, because BAB directly affected the indicator dye, even in the absence of cells. The NTML screening suggested that BAB influenced energy metabolism in bacteria. This was in line with cell metabolism assays, which suggested that BAB inhibited mitochondrial metabolism completely in HepG2 cells. Although the cytotoxicity of BAB precludes its potential to be a clinical antibiotic, it is still interesting to study its targets in more detail, because this might result in identification of new targets for antibiotic discovery and this might help to better understand BAB’s mechanism of inhibiting cancer cell growth.

We believe that there are more interesting fungi producing antimicrobial agents in our fungal library. In the analysis of positive hits, we made a selection and did not cover all of the hits from the initial screening. Among the remaining hits were many strains that are known producers of antimicrobials. Whereas these strains were not selected for further analysis, these are still interesting for further studies and have a chance to produce novel antimicrobials. Actually, much work is still being done with the genera of *Aspergillus* or *Penicillium*, and recently new antimicrobial compounds were identified from them^41–44^. In addition, many potential antimicrobial producing fungi may not have scored positive in our initial screen. These may have gone unnoticed due to low production of active compound, i.e. at levels below MIC. There are options to further screen these fungi, such as to enhance the yield of secondary metabolites by plate extractions instead of liquid–liquid extractions (cf. Figs. 2, 4, S1), to optimize the growth conditions for secondary metabolite production, or to activate silent gene clusters that often encompass the genes encoding the enzymes that produce secondary metabolites by co-culturing with histone deacetylase inhibitors, such as suberoylanilide hydroxamic acid or anacardic acid^45,46^.

To conclude, we found 280 fungal strains with antimicrobial activity among a library of 10,207 fungi and subsequently identified 17 structurally distinct compounds from 26 strains out of the 56 fungi that were selected for further analysis. This indicates that our screening strategy worked for antimicrobial discovery and our fungal collection is a promising source for bioactive compounds. Among the identified compounds, one antimicrobial agent, BAB, was an interesting compound with unknown MoA. We found that BAB treatment affected energy metabolism in both prokaryotic and eukaryotic cells.

## Supplementary Information

Supplementary Information.
