# Supplementary material for: Berkchaetoazaphilone B has antimicrobial activity and affects energy metabolism
Source: Sci Rep. 2021 Sep 21;11:18774. doi: 10.1038/s41598-021-98252-w (PMC8455593; doi:10.1038/s41598-021-98252-w)
Supplement: Supplementary file 1 — Supplementary Information. [file 41598_2021_98252_MOESM1_ESM.pdf]

Supplementary material

Title: Berkchaetoazaphilone B has antimicrobial activity and affects energy metabolism

Xudong Ouyang, Jelmer Hoeksma, Gisela van der Velden, Wouter Beenker, Maria H. van Triest, Boudewijn M.T. Burgering and Jeroen den Hertog.

Supplementary Table S1. Pathogenic bacteria used in this study.

Supplementary Table S2. MID of fungal extracts under different growth conditions of liquid culture.

Supplementary Table S3. Assignments NMR-shifts + HMBC and COSY couplings for BAB.

Supplementary Table S4. MICs of BAB on pathogenic bacteria.

Supplementary Figure S1. Plate extraction profiles of fungus *Monochaetia lutea*.

Supplementary Figure S2. NMR data of berkchaetoazaphilone B.

Supplementary Figure S3. Chemical structures of the antimicrobial compounds identified from this screen.

Supplementary Figure S4. Effect of BAB on membrane potential.

Supplementary Figure S5. Schematics on metabolism of energy.

Supplementary Note. Analytical chemical data of identified compounds.

Table S1. Pathogenic bacteria used in this study.

Strain	For initial screening	For BAB assay
Acinetobacter baumannii 1179 [a]		✓
Acinetobacter calcoaceticus [a]	✓	
Acinetobacter nosocomialis 14-8211 [a]		✓
Enterobacter cloacae complex MC04842 [a]		✓
Enterococcus faecium GV15A623 [a]		✓
Enterococcus faecium VRE GV16D030 [a]	✓	✓
Escherichia coli TEM-3 GVJS004 [a]	✓	✓
Klebsiella pneumoniae SHV-18 GVJS006 [a]	✓	✓
Listeria monocytogenes GV21-4a [a]		✓
Pseudomonas aeruginosa ATCC57853 [b]	✓	✓
Staphylococcus aureus MRSA [a]	✓	
Staphylococcus aureus MSSA 476 GVS0101 [a]		✓
Staphylococcus aureus MRSA USA300 [a]	✓	
Staphylococcus epidermidis GV08A1071 [a]		✓
Stenotrophomonas maltophilia GV20A226 [a]		✓
Streptococcus pneumoniae 05A396 [a]		✓

[a] Gift from University Medical Center Utrecht;

[b] ATCC strains.

Table S2. MID of fungal extracts under different growth conditions of liquid culture*.

Variable	SD[a]	TEMP[b]	OC[c]	GM[d]	GM	IP[e]	IP	IP
**Temperature**	25 °C	15 °C	25 °C	25 °C	25 °C	25 °C	25 °C	25 °C
**Shaking**	No	No	Yes	No	No	No	No	No
**Inoculating plate[f]**	MEA	MEA	MEA	MEA	MEA	OA	CMA	PDA
**Growth medium[g]**	CDB+YE	CDB+YE	CDB+YE	MEB	CDB	CDB+YE	CDB+YE	CDB+YE
Pleurostomophora richardsiae	10	0	0	10	10	10	10	10
Clonostachys compactiuscula	10	10	10	160	20	20	20	20
Monochaetia lutea	40	10	10	40	20	80	80	80
Cristaspora arxii	20	0	0	20	0	20	20	20

* Numbers indicate the Maximum Inhibitory Dilution (MID) of fungal extracts that inhibit the growth of *B. subtilis*. Numbers were repetitive.

a= Standard, b= Temperature, c= Oxygen content, d= Growth medium, e= Inoculating plate

f. MEA= Malt Extract Agar; OA= Oat Agar; CMA= Cornmeal Agar; PDA= Potato Dextrose Agar.

g. CDB= Czapek Dox Broth; YE= Yeast Extract; MEB= Malt Extract Broth.

Table S3. Assignments NMR-shifts + HMBC and COSY couplings for BAB.

berkchaetoazaphilone B (DMSO-d_6)						
	DMSO-d_6		CDCl$_3$			
#	δC[a]	δH[b]	δC[c]	δH[d]	HMBC[b]	COSY[b]
1	148.4	7,69 (s)	147.8	7.48 (s)	3, 4a, 8, 8a	-
3	161.9	-	161.1	-	-	-
4	108.4	6.41 (s)	108.3	6.14 (s)	3, 5, 8a, 10	-
4a	145.6	-	145.3	-	-	-
5	104.7	5.37 (s)	105.2	5.41 (s)	7, 8a	-
6	188.5	-	189.4	-	-	-
7	84.1	-	84.1	-	-	-
8	72.4	-	72.1	-	-	-
8a	107.4	-	107.7	-	-	-
9	19.0	1.46 (s)	18.7	1.61 (s)	6, 7, 8	-
10	42.9	2.48 (m)	42.7	2.56 (m)	3, 4, 11, 12	11
11	64.5	3.91 (m)	65.5	4.18 (m)	3	10,12
12	23.9	1.11 (d)	23.7	1.32 (d)	10, 11	11
13	166.5	-	165.5	-	-	
14	66.8	-	66.8	-	-	
15	197.0	-	195.7	-	-	
16	41.6	2.90 (m)<br>2.75 (m)	42.0	2.84 (m)<br>2.62 (m)	15, 17, 18-21[e]	17
17	23.0	1.41 (t)	23.2	1.50 (m)	15, 16, 18-21[e]	16
18	28.7	1.17[e]	28.9	1.20[e]	[e]	[e]
19	29.1	1.17[e]	29.2	1.28[e]	[e]	[e]
20	29.2	1.17[e]	29.2	1.28[e]	[e]	[e]
21	29.2	1.17[e]	29.3	1.28[e]	[e]	[e]
22	31.7	1.21[e]	31.8	1.23[e]	[e]	[e]
23	22.6	1.23[e]	22.6	1.28[e]	22	24
24	14.4	0.85 (t)	14.1	0.87 (t)	22, 23	23

[a]= measured at 100 MHz, [b]= measured at 400 MHz, [d]= measured at 150 MHz, [d]= measured at 600 MHz, [e]= overlapping signals

Table S4. MICs of BAB on pathogenic bacteria. MICs of BAB on different bacteria were tested starting at a 400 mg/L which was then serially diluted with a factor 2.

Strain	Gram	MIC (mg/L)
Acinetobacter baumannii 1179	-	> 400
Acinetobacter nosocomialis 14-8211	-	> 400
Enterobacter cloacae complex MC04842	-	> 400
Escherichia coli TEM-3 GVJS004	-	> 400
Klebsiella pneumoniae SHV-18 GVJS006	-	> 400
Pseudomonas aeruginosa ATCC57853	-	> 400
Stenotrophomonas maltophilia GV20A226	-	> 400
Enterococcus faecium VRE GV16D030	+	200
*Enterococcus faecium*GV15A623	+	100
Listeria monocytogenes GV21-4a	+	100
Staphylococcus aureus MSSA 476 GVS0101	+	50
Staphylococcus epidermidis GV08A1071	+	200
Streptococcus pneumoniae 05A396	+	50

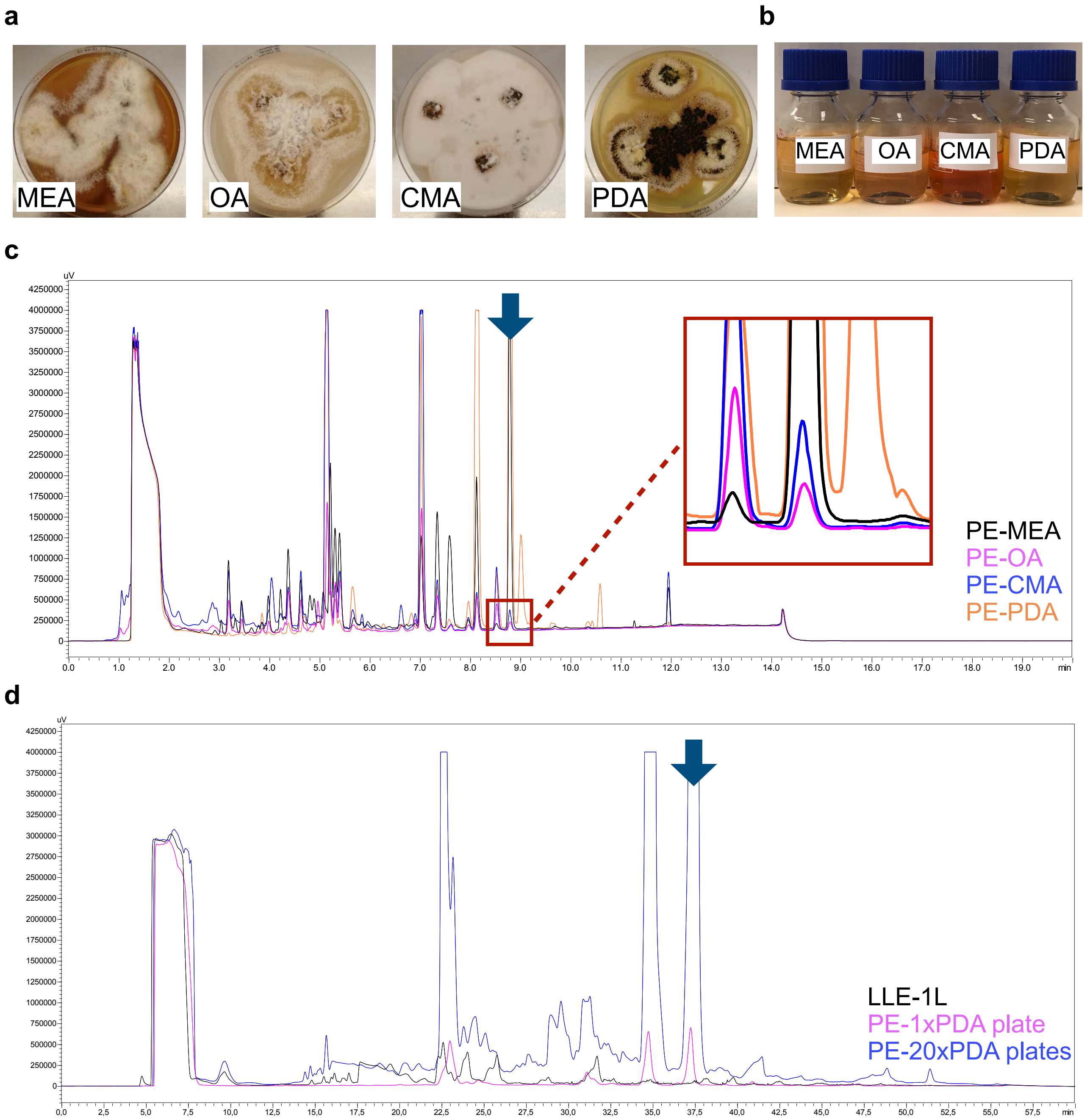

Fig. S1. Plate extraction profiles of fungus *Monochaetia lutea*. Fungus *M. lutea* was cultured on different kinds of agar (a) and extracted by plate extraction (PE) using ethyl acetate (b). The ethyl acetate extracts were then concentrated 1,000 times and analyzed on analytical HPLC (c). The comparison of liquid-liquid extraction (LLE) from 1L culture and PE from both 1 plate and from 20 plates is shown in (d). Arrow indicates active fraction, which was identified to be rugulosin A.

a

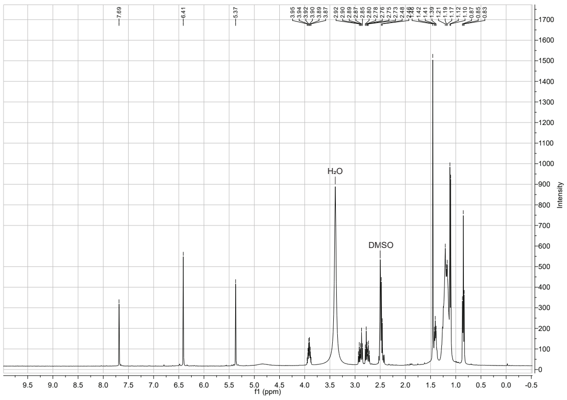
H₂O
DMSO
f1 (ppm)
Intensity

b

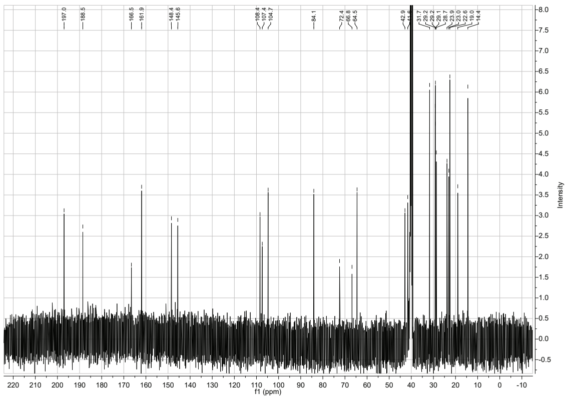
f1 (ppm)
Intensity

c

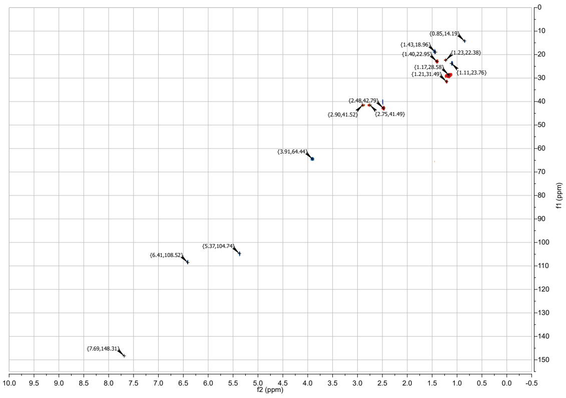
f2 (ppm)
f1 (ppm)

d

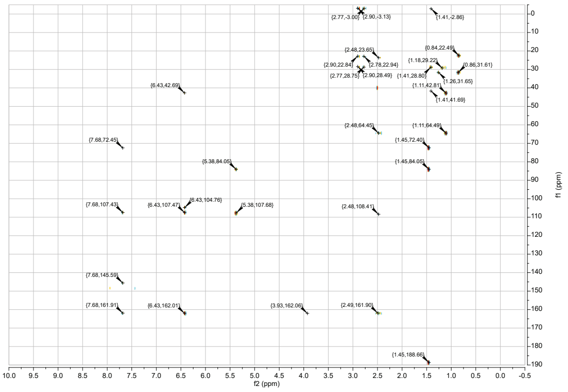
f2 (ppm)
f1 (ppm)

e

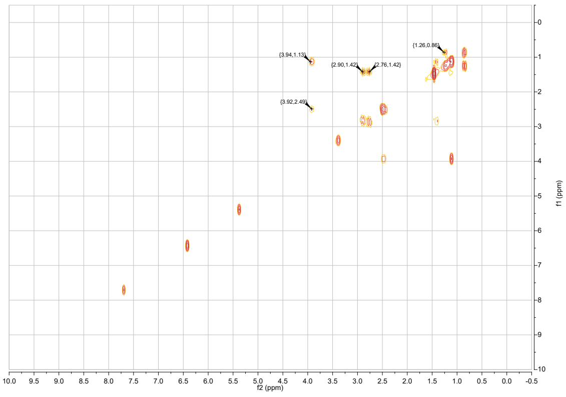
f2 (ppm)
f1 (ppm)

f

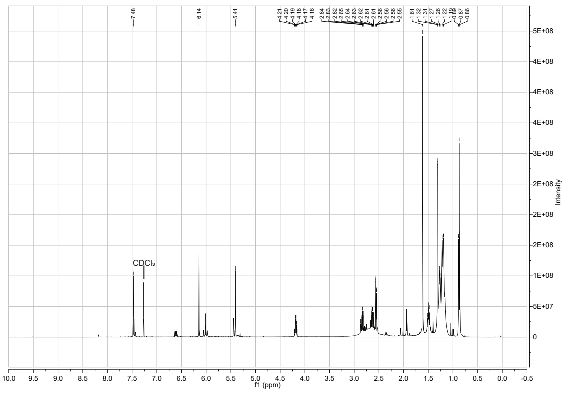
CDCl₃
f1 (ppm)
Intensity

g

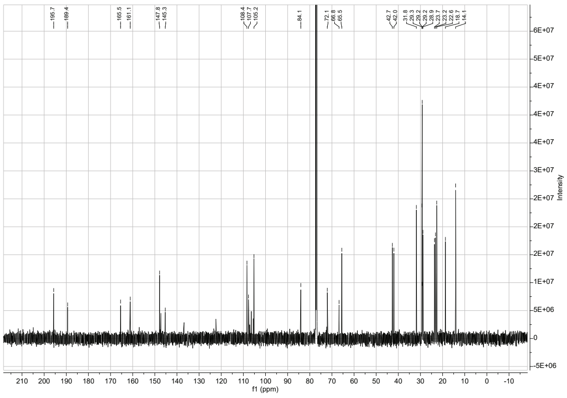
f1 (ppm)
Intensity

Fig. S2. NMR data of berkchaetoazaphilone B. (a) ^{1}H-NMR spectrum, 400 MHz, DMSO-d_6. (b) ^{13}C-NMR spectrum, 100 MHz, DMSO-d_6. (c) HSQC-NMR spectrum, 400 MHz, DMSO-d_6. (d) HMBC-NMR spectrum, 400 MHz, DMSO-d_6. (e) COSY-NMR spectrum, 400 MHz, DMSO-d_6. (f) ^{1}H-NMR spectrum, 600 MHz, $CDCl_3$. (g) ^{13}C-NMR spectrum, 150 MHz, $CDCl_3$.

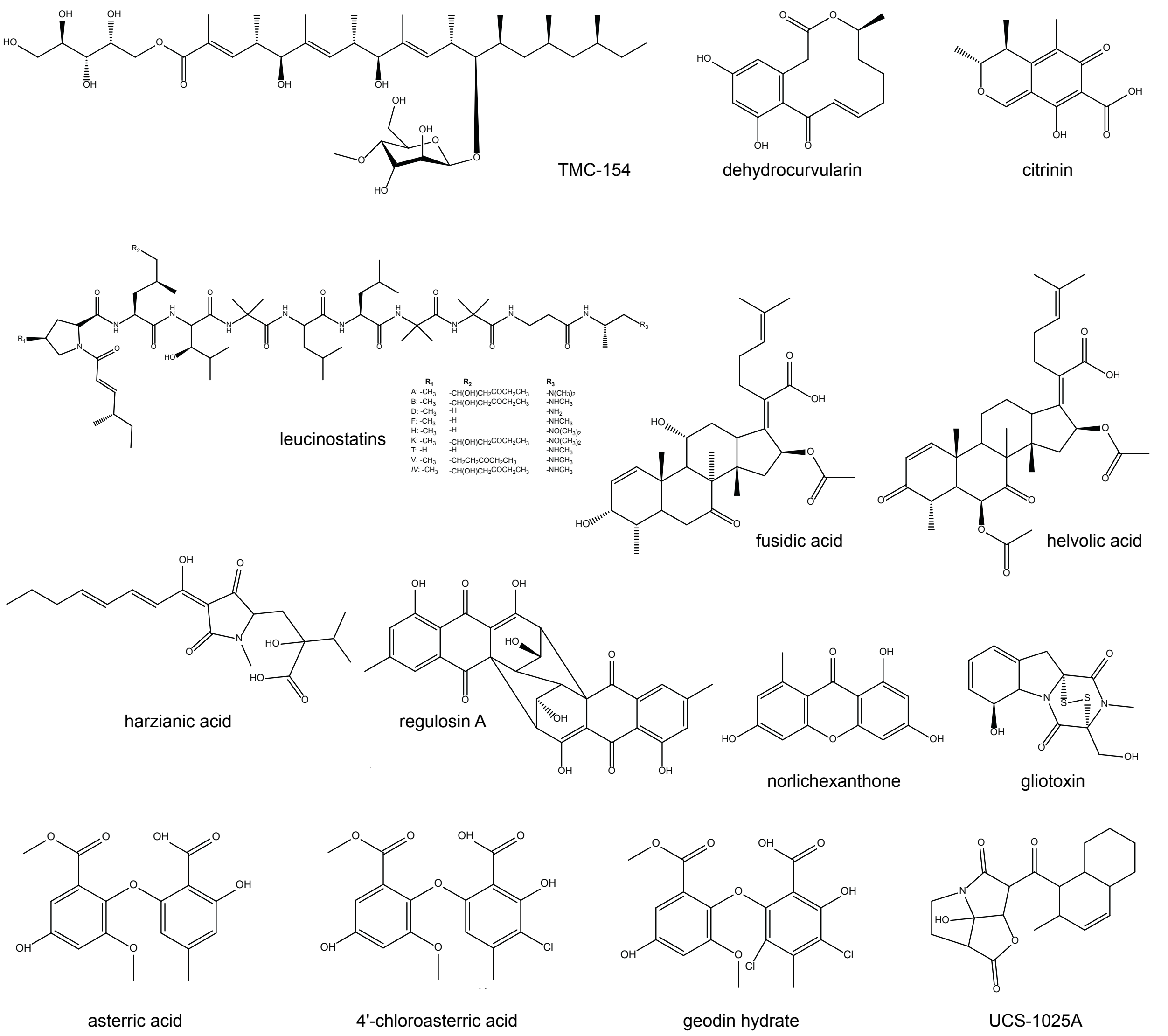

Fig. S3. Chemical structures of the antimicrobial compounds identified from this screen.

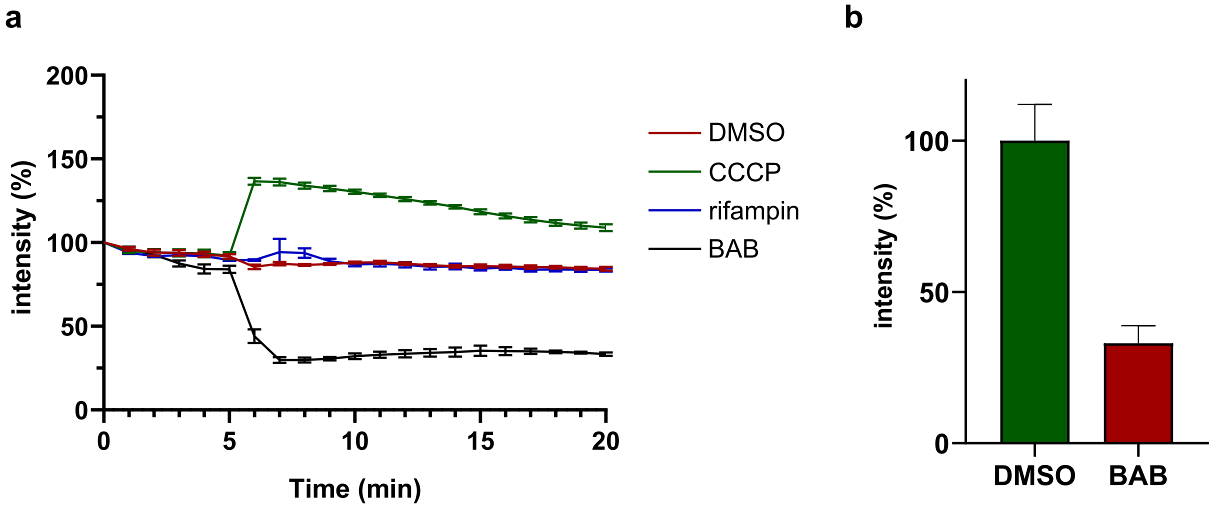

Fig. S4. Effect of BAB on membrane potential. Cell depolarization assay using DiSC$_3$(5) dye. (a) *B. subtilis* membrane potential levels were quantified using the fluorescent dye DiSC$_3$(5). Antimicrobials (as indicated) or DMSO were added after 5 min. The fluorescence is depicted as percentage of the value at the start (t = 0min) over time (min). The mean from biological triplicates is plotted with error bars representing the SEM. Treatment with CCCP (positive control) resulted in an increase in signal due to release of the dye in the medium. Rifampin and DMSO (solvent control) did not affect fluorescence. Surprisingly, BAB treatment resulted in a decrease in signal. (b) Measurements of fluorescence intensity of DiSC$_3$(5) in cell-free LB media showed direct, effect of BAB on DiSC$_3$(5) fluorescence in the absence of cells to the same extent as in (a). The mean from technical triplicates is plotted with error bars representing the SEM.

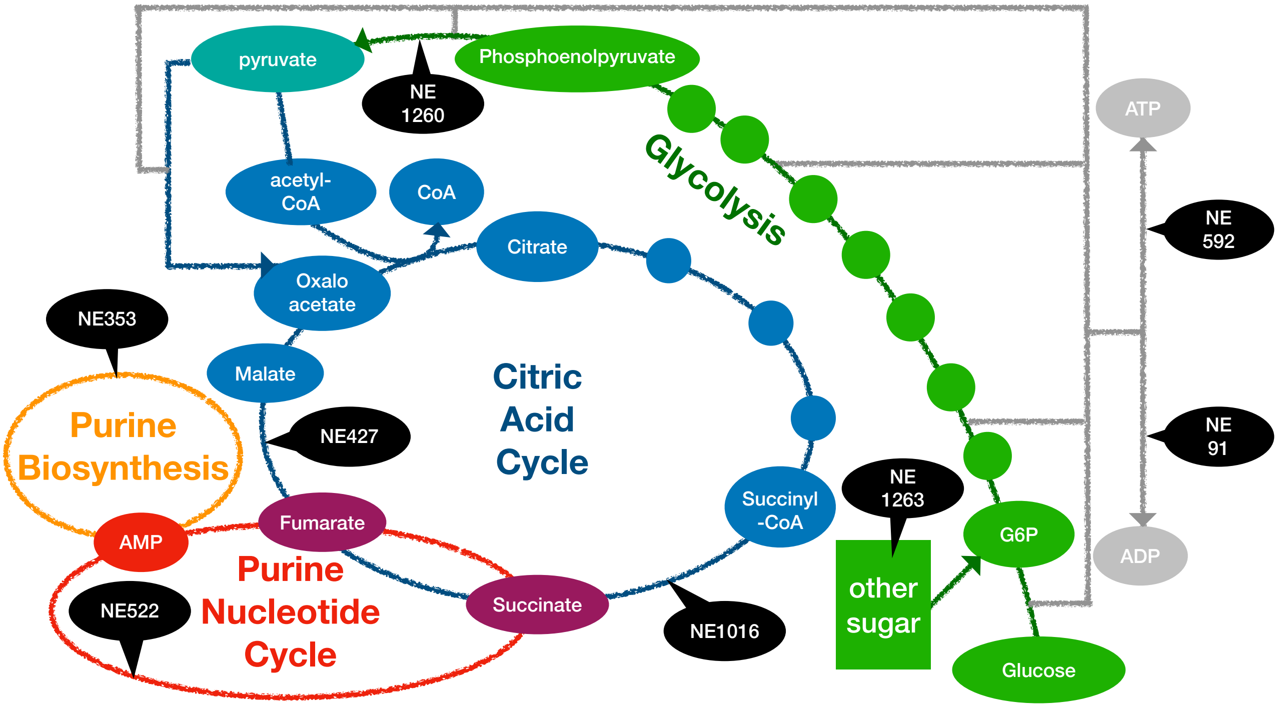

Fig. S5. Schematics on metabolism of energy. The correlation of glycolysis (green), citric acid cycle (blue), purine nucleotide cycle (red) and purine biosynthesis (orange) was listed in the scheme. The processes that involved ATP and ADP transformation were pointed in grey. The processes that NTML hits were involved were pointed in black.

Supplementary notes

Anthracobic acid A: See the paper from Hoeksma et al.[1] for details.

Citrinin: $C_{13}H_{14}O_5$. HRMS: found 273.0706 (M+Na), calculated 273.0739 for $C_{13}H_{14}O_5Na$. ^{1}H-NMR (600MHz, DMSO-d_6): δ= 15.41 (s, OH); 8.63 (s, 1H); 5.00 (dd, *J*= 6.6 Hz, 1H); 3.60 (s, 1H); 3.22 (dd, *J*= 7.2 Hz, 1H); 1.98 (s, 3H); 1.27 (d, *J*= 6.7 Hz, 3H); 1.14 (d, *J*= 7.2 Hz, 3H). ^{13}C-NMR (150MHz, DMSO-d_6): δ= 183.0; 176.9; 174.6; 167.4; 141.5; 121.9; 106.9; 99.8; 82.6; 34.0; 18.5; 18.0; 9.5. UV-Vis λ_{max}: 236 nm, 332 nm. Data consistent with data published by Nielsen and Smedsgaard[2], Barber et al.[3] and Poupko et al.[4].

Asterric acid: $C_{17}H_{16}O_8$. HRMS: found 371.0734 (M+Na), calculated 371.0743 for $C_{17}H_{16}O_8Na$. ^{1}H-NMR (600MHz, DMSO-d_6): δ= 12.98 (bs, 1H); 11.21 (bs, 1H); 9.89 (s, 1H); 6.79 (s, 1H); 6.78 (s, 1H); 6.34 (s, 1H); 6.67 (s, 1H); 3.70 (s, 3H); 3.62 (s, 3H); 2.07 (s, 3H). ^{13}C-NMR (150 MHz, DMSO-d_6): δ= 170.3; 165.6; 160.4; 159.0; 155.6; 153.8; 143.7; 134.4; 125.8; 110.3; 108.0; 105.4; 105.2; 104.5; 56.5; 52.5; 21.9. UV-Vis λ_{max}: 209 nm, 248 nm (sh), 315 nm. Data consistent with data published by Nielsen and Smedsgaard[2] and Liu et al.[5]. Confirmed by commercially available compound.

Dehydrocurvularin: $C_{16}H_{18}O_5$. LCMS (ESI): 291.1 (M+H). ^{1}H-NMR (600MHz, DMSO-d_6): δ= 6.37 (m, 1H); 6.32 (s,1H); 6.24 (s, 2H); 4.74 (m, 1H); 3.38 (dd, *J*= 32.9, 15.6 Hz, 2H); 2.29, 2.18 (m, 2H); 1.82, 1.42 (m, 2H); 1.75, 1.46 (m,

2H); 1.11 (d, *J*= 6.4 Hz. 3H). ^{13}C-NMR (150MHz, DMSO-d_6): δ= 198.1; 170.8; 159.5; 157.9; 154.3; 134.2; 133.0; 118.5; 110.0; 101.9; 72.6; 40.0; 33.8; 33.2; 24.3; 20.4. UV-Vis λ_{max}: 201 nm, 225 nm, 294 nm, 330 nm (sh). Data consistent with data published by Nielsen and Smedsgaard[2] and Kumar et al.[6].

Geodin hydrate: $C_{17}H_{14}Cl_2O_8$. HRMS: found 438.9919 (M+Na), calculated 438.9963 for $C_{17}H_{14}Cl_2O_8Na$. ^{1}H-NMR (600MHz, DMSO-d_6): δ= 9.66 (s, OH); 6.70 (d, *J*= 2.8 Hz, 1H); 6.61 (d, *J*= 2.8 Hz, 1H); 3.63 (s, 3H); 3.54 (s, 3H); 2.43 (s, 3H). ^{13}C-NMR (150 MHz, DMSO-d_6): δ= 165.4; 163.6; 154.3; 152.6; 151.2; 150.6; 136.8; 136.7; 124.3; 116.3; 116.2; 113.5; 107.5; 105.2; 56.7; 52.3; 18.6. UV-Vis λ_{max}: 215 nm, 323 nm. Data consistent with data published by Nielsen and Smedsgaard[2] and Liu et al.[5]. Confirmed by commercially available compound.

4'-Chloroasterric acid: $C_{16}H_{13}ClO_8$. HRMS: found 405.0327 (M+Na), calculated 405.0353 for $C_{17}H_{15}ClO_8Na$. ^{1}H-NMR (600MHz, DMSO-d_6): δ= 9.93 (s, 1H); 6.80 (d, *J*= 2.8 Hz, 1H); 6.79 (d, *J*= 2.8 Hz, 1H); 5.90 (s, 1H); 3.71 (s, 3H); 3.63 (s, 3H); 2.17 (s, 3H). ^{13}C-NMR (150MHz, DMSO-d_6): 171.1; 165.2; 157.6; 156.9; 155.7; 153.6; 142.0; 134.2; 125.4; 113.7; 108.2; 106.7; 105.3; 104.5; 56.5; 52.4; 20.9. UV-Vis λ_{max}: 208 nm, 249 nm (sh), 321 nm. Data consistent with data published by Liu et al.[5].

Fusaric acid: See the paper from Hoeksma et al.[1] for details.

Fusidic acid: $C_{31}H_{48}O_6$. HRMS: found 539.3337 (M+Na), calculated 539.3349 for $C_{31}H_{48}O_6$. UV-Vis λ_{max}: 200 nm, 220 (sh) nm. Data consistent with data published by Nielsen and Smedsgaard[2]. Confirmed by commercially available compound.

Gliotoxin: $C_{13}H_{14}N_2O_4S_2$. HRMS: found 327.0490 (M+H), calculated 327.0490 for $C_{13}H_{15}N_2O_4S_2$. ^{1}H-NMR (600MHz, DMSO-d$_6$): δ= 6.03 (m, 1H); 5.97 (m, 1H); 5.65 (d, 1H, *J*= 9.7 Hz); 4.86 (m, 1H); 4.55 (d, *J*= 13.1 Hz, 1H); 4.34 (d, *J*= 12.7 Hz, 1H); 4.23 (d, *J*= 12.7 Hz, 1H); 4.07 (d, *J*= 11.4 Hz), 3.74 (d, *J*= 11.4 Hz) (1H); 3.64 (m, 1H); 3.11 (s, 3H); 3.00 (s, 1H). ^{13}C-NMR (150MHz, DMSO-d$_6$): δ= 166.0; 164.6, 133.3, 130.1; 124.1; 119.3; 78.8; 76.3; 73.2; 69.8; 59.2; 36.3; 28.1. UV-Vis λ_{max}: 200 nm, 268 nm. Data consistent with data published by Kaouadji et al[7] and Sun et al[8].

Harzianic acid: $C_{19}H_{27}NO_6$. HRMS: found 388.1750 (M+Na), calculated 388.1736 for $C_{19}H_{27}NO_6Na$. Elemental composition analyses: C 61,4%; O 21,8%; H 7,0%; N 3,8%. ^{1}H-NMR (600 MHz, CDCl$_3$): δ= 7.55 (m, 1H); 7.00 (d, *J*= 15.1 Hz, 1H); 6.38 (m, 1H), 6.37 (m, 1H); 3.63 (dd, *J*= 10.6, 1,0 Hz, 2H); 2.97 (s, 3H); 2.48-1.89 (d, 2H); 2.24 (dd, 2H); 2.02 (m, 1H); 1.50 (m, 2H); 0.99 (m, 3H); 0.99 (m, 3H); 0.95 (t, 3H). ^{13}C-NMR (150 MHz, CDCl$_3$): δ= 197.3; 176.7; 176.3; 173.2; 149.9; 147.6; 129.6; 119.1; 99.7; 79.9; 64.1; 36.0; 35.5; 33.8; 26.6; 21.8; 17.5; 16.2; 13.7. UV-Vis λ_{max}: 244 nm, 363 nm. Data consistent with data published by Sawa et al[9].

Helvolic acid: $C_{33}H_{44}O_8$. HRMS: found 595.3248 (M+Na), calculated 595.3248 for $C_{33}H_{44}O_8Na$. ^{1}H-NMR (400 MHz, $CDCl_3$): δ= 7.33; 5.87; 5.25; 5.10; 2.78; 2.63; 2.45; 2.26; 2.11; 1.96; 1.69; 1.59; 1.44; 1.27; 1.18; 0.96. UV-Vis λ_{max}: 202 nm, 232 nm. Data consistent with data published by Nielsen and Smedsgaard[2] and Tschen et al.[10].

<u>Leucinostatins:</u>

Leucinostatin A: $C_{62}H_{111}N_{11}O_{13}$. LCMS (ESI+): 1218.9 (M+H). MS-MS fragmentation: 1218.9 (base), 960.9, 875.8, 762.7, 649.6, 631.6, 564.5, 546.5, 435.2, 222.2. UV-Vis λ_{max}: 222 nm.

Leucinostatin B: $C_{61}H_{109}N_{11}O_{13}$. LCMS (ESI+): 1205.4 (M+H). MS-MS fragmentation: 1205 (base), 960.9, 875.8, 762.7, 649.6, 631.6, 564.5, 546.5, 435.4, 222.2.

Leucinostatin D: $C_{57}H_{103}N_{11}O_{11}$. LCMS (ESI+): 1119.1 (M+H). MS-MS fragmentation: 1119 (base), 860.9, 775.8, 662.7, 549.5, 531.5, 464.5, 446.5, 335.3, 222.2.

Leucinostatin F: $C_{56}H_{101}N_{11}O_{11}$. LCMS (ESI+): 1105.7 (M+H). MS-MS fragmentation: 1105 (base), 860.9, 775.8, 662.7, 549.5, 531.5, 464.5, 446.5, 335.3, 222.2.

Leucinostatin H: $C_{57}H_{103}N_{11}O_{12}$. LCMS (ESI+): 1135.5 (M+H). MS-MS fragmentation: 1135 (base), 860.9, 775.8, 662.7, 549.5, 531.5, 464.5, 446.5, 335.3, 222.2.

Leucinostatin K: $C_{62}H_{111}N_{11}O_{14}$. LCMS (ESI+): 1235.2 (M+H). MS-MS fragmentation: 1234.8 (base), 960.9, 875.8, 762.7, 649.6, 631.6, 564.5, 546.5, 435.4, 222.2.

Leucinostatin T: $C_{55}H_{99}N_{11}O_{11}$. LCMS (ESI+): 1091.7 (M+H). MS-MS fragmentation: 1091 (base), 846.8, 761.7, 648.6, 535.5, 517.5, 450.4, 432.4, 321.3. 208.2.

Leucinostatin V: $C_{61}H_{109}N_{11}O_{12}$. LCMS (ESI+): 1189.1 (M+H). MS-MS fragmentation: 1189 (base), 944.9, 859.9; 746.8, 633.6, 615.6, 548.6, 530.6, 419.4, 222.2.

Leucinostatin *IV*: $C_{61}H_{111}N_{11}O_{12}$. LCMS (ESI+): 1190.4 (M+H). MS-MS fragmentation: 1191 (base), 946.8, 875.8, 762.7, 649.6, 631.6, 617.5, 564.5, 546.5, 435.4, 222.2. Data consistent with data published by Isogai et al.[11] and Martinez and Morales[12].

Norlichexanthone: $C_{14}H_{10}O_5$. HRMS: found 259.0619 (M+H), calculated 259.0606 for $C_{14}H_{11}O_5$. ^{1}H-NMR (300 MHz, DMSO-d_6): δ= 6.66 (1H); 6.52 (1H); 6.28 (1H); 6.13 (1H); 2.73 (3H). Data consistent with data published by Nielsen and Smedsgaard[2] and Kawakami et al.[13].

Rugulosin A: $C_{30}H_{22}O_{10}$. HRMS: found 543.1292 (M+H), calculated 543.1291 for $C_{30}H_{23}O_{10}$. LCMS (ESI): 543.2 (M+H). ^{1}H-NMR (600 MHz, DMSO-d_6): δ= 14.73 (bs, OH); 11.41 (s, OH); 7.48 (d, *J*= 0.9 Hz, 1H); 7.22 (s, 1H); 4.41 (m,

1H); 3.39 (s, 1H); 2.81 (d, *J*= 6.0 Hz, 1H); 2.45 (s, 3H). ^{13}C-NMR (150 MHz, DMSO-d_6): δ= 194.4; 186.5; 181.1; 160.6; 148.1; 132.5; 124.5; 121.0; 114.6; 106.6; 69.0; 58.8; 56.1; 48.2; 21.9. UV-Vis λ_{max}: 250 nm, 392 nm. Data consistent with data published by Yamazaki et al[14].

TMC-154: $C_{41}H_{72}O_{14}$. HRMS: found 811.4790 (M+Na), calculated 811.4820 for $C_{41}H_{72}O_{14}$Na. ^{1}H-NMR (600 MHz, DMSO-d_6): δ= 6.69 (dd, *J*= 14.8, 7.2Hz, 1H); 5.26 (d, *J*= 8.6 Hz, 1H); 5.21 (d, *J*= 8.6 Hz, 1H); 5.12 (d, *J*= 9.6 Hz, 1H); 4.86 (m, 1H); 4.55 (m, 1H); 4.16 (s, 1H); 3.88 (d, *J*= 8.2 Hz, 1H); 3.85^{a*}; 3.67^{a*}; 3.61^{a*}; 3.59^{a*}; 3.53^{a*}; 3.44 (s, 1H); 3.43(s, 1H); 3.41 (s, 1H); 3.33 (d, *J*= 3.1 Hz, 1H); 3.31 (d, *J*= 2.8 Hz, 1H); 3.12 (m, 1H) ; 2.89 (m, 1H); 2.57 ^{a*}; 2.56 ^{a*}; 2.50 ^{a*}; 2.43 ^{a*}; 1.81 (s, 3H); 1.58 (s, 3H); 1,57 (s, 3H); 1.53 (s, 3H); 1.28^{a}; 1.21-1.11 (2H) ^{a*}; 1.20 ^{a*}; 0.92 (d, *J*= 6.5 Hz, 3H); 0.82 ^{a*}; 0.79 ^{a*}; 0.78 ^{a*}; 0.72 (d, *J*= 6.3 Hz, 3H). ^{13}C-NMR (150 MHz, DMSO-d_6)*: δ= 167.4, 146.5, 138.2, 136.7, 136.1, 131.8, 131.1, 130.3, 127.3, 96.5, 86.2, 81.6, 81.2, 77.5, 76.9, 74.4, 73.4, 71.6, 70.2, 68.0, 64.0, 63.2, 61.3,60.0, 44.3, 37.4, 36.4, 34.4, 32.5, 30.2, 29.7, 21.9, 19.1, 17.8, 16.9, 12.9, 11.6, 11.6, 11.5. ^{a}= no multiple assignment due to overlapping peaks, *=tentative assignments based on HSQC and HMBC-NMR. UV-Vis λ_{max}: 195 nm, 227 nm (sh). Data consistent with data published by Kohno et al[15].

UCS-1025A $C_{20}H_{25}NO_5$ HRMS: found 382.1623 (M+Na), calculated 382.1630 for $C_{20}H_{25}NO_5$Na. ^{1}H-NMR (500 MHz, $CDCl_3$): δ= 5.57 (m, 1H); 5.40 (d, *J*= 9.9 Hz, 1H); 4.73 (s, 1H); 4.06 (s, 1H); 3.88, 3.36 (m, 2H); 3.25 (d, *J*= 8.9 Hz 1H);

3.18 (dd, J= 11.2, 5,3 Hz, 1H); 2.90 (d, 1H), 2.77 (m, 1H); 2.56 (m, 1H); 1.76 (m, 5H); 1.51 (m, 1H); 1.32 (m, 2H); 1.07 (m, 1H); 0.88 (m, 1H); 0.78 (d, J= 7.0 Hz, 3H). ^{1}H-NMR (100 MHz, $CDCl_3$): δ= 209.2; 173.8; 166.2; 130.6; 130.3; 100,5; 80.2; 66.3; 58.8; 47.6; 42.1; 41.8; 36.7; 32.8; 30.1; 29.9; 29.8; 26.5; 26.4; 17.6. UV-Vis λ_{max}: 202 nm, 262 nm. Data consistent with data published by Mizukami et al[16], Nikai et al[17] and Agatsuma et al[18].

References

1. Hoeksma, J. *et al.* A new perspective on fungal metabolites: identification of bioactive compounds from fungi using zebrafish embryogenesis as read-out. *Sci. Rep.* **9**, 1–16 (2019).
2. Nielsen, K. F. & Smedsgaard, J. Fungal metabolite screening: Database of 474 mycotoxins and fungal metabolites for dereplication by standardised liquid chromatography-UV-mass spectrometry methodology. *J. Chromatogr. A* **1002**, 111–136 (2003).
3. Barber, J., Cornford, J. L., Howard, T. D. & Sharples, D. The structure of citrinin in vivo. *J. Chem. Soc. Perkin Trans. 1* **2**, 2743–2744 (1987).
4. Poupko, R., Luz, Z. & Destro, R. Carbon-13 NMR of citrinin in the solid state and in solutions. *J. Phys. Chem. A* **101**, 5097–5102 (1997).
5. Liu, D. *et al.* Diphenyl derivatives from coastal saline soil fungus Aspergillus iizukae. *Arch. Pharm. Res.* **38**, 1038–1043 (2015).
6. Ganesh Kumar, C. *et al.* Metabolite profiling and biological activities of bioactive compounds produced by Chrysosporium lobatum strain BK-3 isolated from Kaziranga National Park, Assam, India. *Springerplus* **2**, 1–9 (2013).
7. Kaouadji, M., Steiman, R., Murandi, F. S., Krivobok, S. & Sage, L. Gliotoxin: Uncommon1h couplings and revised1h- and13c-nmr assignments. *J. Nat. Prod.* **53**, 717–719 (1990).

8. Sun, Y. *et al.* Gliotoxin analogues from a marine-derived fungus, penicillium sp., and their cytotoxic and histone methyltransferase inhibitory activities. *J. Nat. Prod.* **75**, 111–114 (2012).

9. Sawa, R. *et al.* Harzianic acid, a new antimicrobial antibiotic from a fungus. *J. Antibiot. (Tokyo).* **47**, 731–732 (1994).

10. Tschen, J. S. M., Chen, L. L., Hsieh, S. T. & Wu, T. S. Isolation and phytotoxic effects of helvolic acid from plant pathogenic fungus Sarocladium oryzae. *Bot. Bull. Acad. Sin.* **38**, 251–256 (1997).

11. Isogai, A., Nakayama, J., Takayama, S., Suzuki, A. & Kusai, A. Structural Elucidation of Minor Components of Peptidyl Antibiotic P168s (Leucinostatins) by Tandem Mass Spectrometry. *Biosci. Biotechnol. Biochem.* **56**, 1079–1085 (1992).

12. Martinez, A. F. C. & Moraes, L. A. B. Liquid chromatography-tandem mass spectrometry characterization of five new leucinostatins produced by Paecilomyces lilacinus CG - 189. *J. Antibiot. (Tokyo).* **68**, 178–184 (2015).

13. Kawakami, H. *et al.* Norlichexanthone produced by cultured endolichenic fungus induced from Pertusaria laeviganda and its antioxidant activity. *Biosci. Biotechnol. Biochem.* **83**, 996–999 (2019).

14. Yamazaki, H., Koyama, N., Oura, S. & Tomoda, H. New rugulosins, Anti-MRSA antibiotics, produced by Penicillium radicum FKI-3765-2. *Org. Lett.*

12, 1572–1575 (2010).

15. Kohno, J. *et al.* TMC-171A, B, C and TMC-154, novel polyketide antibiotics produced by Gliocladium sp. TC 1304 and TC 1282. *J. Antibiot. (Tokyo).* **52**, 1114–1123 (1999).

16. Mizukami, T. *et al.* Furoindolizines, EP0849267B, European Patent Office. (2001).

17. Nakai, R. *et al.* UCS1025A, a novel antibiotic produced by Acremonium sp. *J. Antibiot. (Tokyo).* **53**, 294–296 (2000).

18. Agatsuma, T. *et al.* UCS1025A and B, New Antitumor Antibiotics from the Fungus Acremonium Species. *Org. Lett.* **4**, 4387–4390 (2002).
